# Multiscalar Temporality in Human Behaviour: A Case Study of Constraint Interdependence in Psychotherapy

**DOI:** 10.3389/fpsyg.2020.01685

**Published:** 2020-08-20

**Authors:** Juan M. Loaiza, Sarah B. Trasmundi, Sune V. Steffensen

**Affiliations:** ^1^Independent Scholar, Colombia, United Kingdom; ^2^Centre for Human Interactivity, Department of Language and Communication, University of Southern Denmark, Odense, Denmark; ^3^Danish Institute for Advanced Study, Odense, Denmark; ^4^Center for Ecolinguistics, South China Agricultural University, Guangzhou, China; ^5^College of International Studies, Southwest University, Chongqing, China

**Keywords:** time scale, temporality, constraint closure, psychotherapy, enactive approach, ecological psychology, distributed language, cognitive ethnography

## Abstract

Ecological psychology (EP) and the enactive approach (EA) may benefit from a more focused view of lived temporality and the underlying temporal multiscalar nature of human living. We propose multiscalar temporality (MT) as a framework that complements EP and EA, and moves beyond their current conceptualisation of timescales and inter-scale relationships in organism-environment dynamical systems. MT brings into focus the wide ranging and meshwork-like interdependencies at play in human living and the questions concerning how agents are intimately entangled in such meshworks, utilising them as resources for skilful living. We develop a conceptual toolkit that highlights temporality: Firstly, we address lived temporality. We use a case study from psychotherapy to show how a person’s skilful engagement with the world is best described as adaptive harnessing of interdependencies of constraints residing across a wide range of timescales. We call this skill time-ranging. Secondly, the case study provides a proof of concept of the integration of an idiographic approach to human conversing and a more general theory of emergent organisation rooted in theoretical biology. We introduce the existing concept of constraint closure from theoretical biology and scale it up to human interactivity. The detailed conceptualisation of constraint interdependencies constitutes the backbone of the proposal. Thirdly, we present a heuristic mapping of what we call organising frames. The mapping guides the conceptualisation of the emergence of inter-scale relationships and serves as an epistemic tool that brings together nomothetic and idiographic approaches. Finally, we combine new ideas with re-interpretations of existing EP and EA concepts and elaborate on the need of a fresh new look at the implicit and sometimes missing conceptualisations of temporality in the EP and EA literature.

## Introduction: Environments, Bodies, and Temporalities

We present a conceptual model of multiscalar temporality (MT), which brings a more nuanced understanding of the temporal dynamics of human living and a richer interpretational frame for both ecological psychology and the enactive approach (hereafter EP and EA, respectively). The model integrates work on temporality from a distributed language perspective (Steffensen and Pedersen, 2014; Uryu et al., 2014; Cowley and Steffensen, 2015) with a view of biological organisation based on the notion of constraint closure (Montévil and Mossio, 2015; Moreno and Mossio, 2015).

We propose that EP and EA may benefit from a more focused formulation of MT. Our proposal constitutes a third perspective in which EP and EA may find some of their commonalities as well as divergences expressed in a new way.

Whereas our proposal highlights the varieties of *temporalities*, EP and EA focus on varieties of *environments* and varieties of *bodies*, respectively. On the one hand, EP, especially in its radical embodied version (Chemero, 2009) together with the skilled intentionality framework (Rietveld and Kiverstein, 2014) provide a more nuanced view of ecological information^1^ that we interpret as an account of the varieties of environments or, more precisely, varieties of ecological information (Bruineberg et al., 2018; Baggs and Chemero, 2019; see also Heft, 2001; Heras-Escribano, 2020). On the other hand, a recent development in EA upgrades the discussion of autonomy and individuation to what Di Paolo calls a “theory of human bodies” (Di Paolo, 2020). EA seeks to substantiate the hypothesis of the continuity of mind and life (Thompson, 2007). According to EA, the continuity consists of the individuation of bodies at various levels – metabolic, sensorimotor, inter-subjective, and linguistic – by virtue of the dialectics between the openness that maximises self-production and the closeness that maximises self-distinction. The idea of a variety of bodies finds its most explicit elaboration in Di Paolo et al. (2018).

The third perspective complements and brings forth some of the missing links between EP and EA, yet it is rooted in a different ground. It is neither based on the preoccupation with ecological information (EP), nor on the question of the individuality of cognitive agents (EA). Instead MT is about the wide ranging and *meshwork-like interdependencies* at play in human living and the questions concerning how agents both are intimately entangled in such meshworks and utilise them as resources for skilful living.

Nevertheless, across the three perspectives we identify a common effort to understand the place of lived experience within relational views of mind and cognition that do justice to essential interdependencies beyond the individual agent. Our starting point is an account of *lived temporality*. We use a case study from psychotherapy to show how a person’s skilful engagement with the world is best described as adaptive harnessing of interdependencies of processes and constraints across a wide range of timescales. The patient in our psychotherapy case study (pseudonymised as “Alice”) experiences a complex here-and-now characterised by an entanglement of fast changes of vocal gesturing, sudden emotional changes and distorted proprioception, together with recurrent negative narratives about herself, and the persistent conflicting relationships with her spouse and parents, the latter characterised by cycles of abuse and neglect. Alice’s experienced tensions in the present, we suggest, can only be understood in the light of a deeper view of interdependencies beyond here-and-now.

Crucially, we show how, as the conversation progresses along several sessions of psychotherapy, the therapist picks up important correspondences between the fast timescales of Alice’s vocal gesturing and the emotional effects of slow timescales of family relationships. Guided by the therapist, Alice is exposed to the tacit interdependencies that permeate the painful tensions experienced in conversation. By doing so, she expands her own capacity to modulate and eventually improve the grip on everyday life situations. We suggest that with the skilful intervention of psychotherapists, patients may develop more flexible and adaptive ways of harnessing and meshing processes and constraints at multiple timescales.

The case study provides a proof of concept of the integration of an idiographic approach to human conversing and a more general theory of emergent biological organisation. As a generic example, the case study is used across sections “From Lived Temporality to Mapping Interdependencies” and “The Backbone: Constraint Interdependence”, demonstrating different aspects of the general framework we propose.

We elaborate on two main themes combining new ideas with re-interpretations of existing EP and EA concepts. Firstly, we address how persons bring forth *lived temporality* as a matter of skilful navigation in a temporally deep present. We unpack the view that persons align and position themselves with respect to multiscalar temporal phenomena – for example, biological cycles, cycles of ritual and social life, and personal relationships. In doing so, we claim, persons maintain the coherence of their lived here-and-now. In section “From Lived Temporality to Mapping Interdependencies”, we introduce the notion of *time-ranging* to account for skilful intentionality,^2^ a concept that highlights the richness of lived temporality. Secondly, we address the nature of the temporal entanglement itself by elaborating on the theme of *organisational interdependence.^3^* We map the actual networks of constraints at play in human real-life situations. To this aim, we use a coarse-grained heuristic distinction of various *organising frames* each with a characteristic temporal depth. The mapping serves initially as an epistemic tool, bringing together nomothetic and idiographic approaches. In section “The Backbone: Constraint Interdependence” we elaborate a fine-grained account of inter-timescale dependencies with a more precise use of the *constraint closure* notation.

In section “Time-Ranging in Psychotherapy: An Ethnographic Investigation”, we return to a fully expanded account of Alice’s case and show how the lens of MT gives us a principled way of understanding the dynamics of human interaction. We demonstrate the applicative perspectives and integrative potential of our model of MT.

Finally, in section “Discussion”, we use these insights to reconsider the relation of our proposal with EP and EA, and conclude that a new third perspective can complement those approaches while addressing their conceptual obscurity with respect to human temporality.

## From Lived Temporality to Mapping Interdependencies

### Time-Ranging Phenomena and Skill

We introduce two complementary terms: *temporal range* and *time-ranging* (Cowley and Steffensen, 2015). Temporal range, in contrast to the more common notion of timescale, captures the range of processes spreading across multiple spatiotemporal scales. A relevant range of timescales must satisfactorily describe a domain of interdependence of some processes and constraints. In our case study, we observe that Alice’s utterances are not so much serial events occurring in a single conversational timescale against the background of some generic or fixed sociolinguistic context. Instead, each of Alice’s vocal gestures presents wide configurations of connections between various converging events. A sudden rise in speech rate during therapy may reveal itself to be connected with the current affective state of the relationship with Alice’s mother, a relationship that stands as a living legacy of childhood neglect. The therapeutic conversation, we argue, is shaped by the widening and narrowing of temporal ranges related to multiple events in Alice’s life.

Time-ranging refers to the performative aspect of managing [i.e., (re-) entangling/disentangling] temporal ranges. Theoretically, it corresponds to the ability to modulate temporal ranges in ways that grant a unique degree of adaptive behaviour when managed skilfully. In everyday life,^4^ persons imbued in their various life-projects rely on time-ranging when exploring past experiences, projecting future activities, and further exploiting sociocultural norms and habits to reduce tensions and improve their experiential grip on the “thick here-and-now”^5^. Persons use utterances and navigate interpersonal coordination in ways that swiftly switch their attention between earlier events and latent projects in a wide trans-situational range (cf. Linell, 2009). Consider for example the social practice of performing non-contemporary music based on music scores. One could see the musical score as a code that dictates the minute movements of the musicians, so that the musicians’ only task is to create a one-to-one conversion of the sheet into an audible format. However, as musicians and audiences are well aware, no two performances are alike. Any performance is a complex social event, and the score is only one constraining element in this event (Loaiza, 2016). To exemplify, consider how a choir performs Allegri’s piece, *Miserere mei, Deus* (ca. 1630), based on one of the biblical psalms.^6^ The skilful performance does not only depend on their ability to coordinate their vocalisations to perform the music, and it does not only depend on their attuning their vocalisations to the coded information in the musical score. Rather, they enact a thick here-and-now that solicits many temporally spread resources. Beyond the vocalists’ here-and-now situated behaviour and Allegri’s score, these resources include slow changing constraints pertaining to the social institution of Church music, and Psalm 51 in the Christian canon, as well as the musical enskilment that the singers have acquired through their training and experience. Somewhere in between these timescales, one finds professional knowledge about various ornamentation techniques in Renaissance polyphony, such as the so-called *abbellimenti*. Thus, years, decades, centuries, and millennia are enfolded within the duration of the piece. Accordingly, the conductor and the choir can meaningfully mould their performance depending on different interpretations of the text, the music, the composer’s intentions, the traditions involved, and even the location of the musical performance. In doing so, they enact their present through multiple temporal ranges, and they give weight to the one or the other of these ranges. In short, they act as *time-rangers*.

As a form of skilful and habitual intentionality, time-ranging is also susceptible to impoverishment. Such an impoverishment comes to the fore in psychopathology, which may be understood as a form of time-ranging where a person cannot escape the affective matrices of past experiences, so that sedimented emotions dominate their present behaviour. In Modell’s (2003, p. 40) succinct formulation, the past invades the present.

Psychotherapy serves as a good case for demonstrating the function and importance of developing time-ranging skills. Time-ranging, when managed successfully, enables personal development, emotional self-regulation, flexible embodied enskilment, and self-reflexivity.^7^ Human living is constrained by multiple temporal ranges, and psychotherapy is an activity where patients are given the opportunity to renew their sensitivity to the interdependencies of processes and constraints within some of those ranges. In psychotherapy, recent events in the patient’s life are explored in relation to how memories and legacies from the patient’s childhood and adolescence become persistently re-enacted in the ecology of personal relationships and narratives.^8^ In our case study, by exploring how Alice’s situated behaviour and emotions relate to past, present, and future events – involving a history with traumatic events, impaired interpersonal relationships, and inabilities to control emotional reactions – Alice is encouraged to develop a repertoire of reflexivity pertaining to her lived experience (we fully unpack the case study in section “Time-Ranging in Psychotherapy: An Ethnographic Investigation”). The goal of psychotherapy is thus to develop a richer cognitive-emotional repertoire that allows for more flexible, adaptive, and reflexive agency in challenging situations.

### How Do We Map an Entangled Whole?

In the process of psychotherapy, we examine the interplay between temporal ranges pertaining to Alice’s self-narrative, the sociality of an interpersonal relation that maintains a legacy of childhood experiences, the therapist’s own expertise as a time-ranger – sedimented through participation in institutional practices – and the basic commonalities of the two individuals’ sensorimotor coupling in conversation. Given this entangled whole, a crucial empirical question remains: how do we map such complex multiscalar interdependencies? Or more specifically, how can we, for example, distinguish between the tight links of Alice’s vocal gesture, mood, and current family situation, and the more generic dynamics of two adult humans coordinating interaction in conversation, without severing interdependencies or reducing the phenomena to narrow and thus uninformative timescales?

**FIGURE 1:**
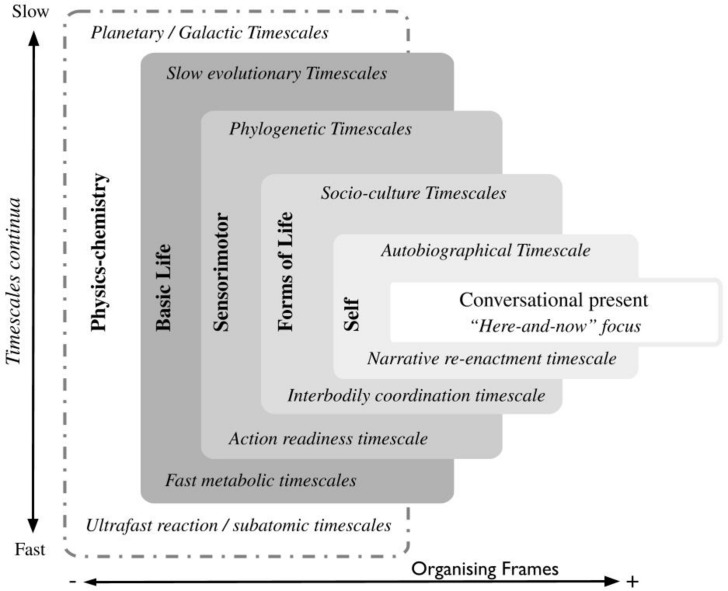
Mapping of organising frames. This is a version of the model in Steffensen and Pedersen (2014); Uryu et al. (2014). In the proposed notation, the horizontal dimension indicates the increase in number of nested frames and degree of specificity. For instance, the conversational present is irreducible to any single organising frame, as it is co-constrained by all the frames (hence its specificity). The vertical dimension indicates a timescale continuum from slower timescales (at the top of the figure) to faster timescales (at the bottom of the figure). Hence, the size of each rectangle represents both the maximum temporal range for each frame and the overlapping of temporal ranges between frames. Sensorimotor, Forms-of-life, and Self frames are step-like narrower subdomains of life. Each subdomain necessarily comprises gradually narrower maxima of timescales. Timescale extrema for each frame are indicated at the top and bottom of each rectangle, for example, the Forms-of-life frame is flanked by “interbodily coordination timescale” and “socio-cultural timescales”, which is read as expressing the fastest and slowest relevant timescales of the Forms-of-life organising frame. The “conversational present” is represented as an elongated rectangle (in the middle of the figure). It represents a nested and emergent organisation of the “here-and-now” constrained by the other five organising frames.

Following a previous model by Steffensen and Pedersen (2014)^9^., we propose a map (Figure 1) that represents a general logic of emergence of (sub)domains of activity that we call *organising frames*.

In the map, smaller frames represent sets of narrower temporal ranges emerging from larger, temporally wider frames. Similar to a cartographic map, its logic is generalisable, but the content of the map, in this case the particular themes of the progression from wider to narrower frames, corresponds to particular research interests. Each frame allows us to make an approximation to a variety of possible boundaries of the interdependencies at play, in our case study, in conversations. Using Alice’s therapy sessions as a template, the map represents the emergence of a focal frame of “conversational present” in which psychotherapy plays out. The conversational present is enabled and emerges from a frame of Self (Alice’s self-narratives), itself emerging from Forms-of-life (Alice’s social life and relationships), Sensorimotor frame (Alice’s bodily habits with respect to an environment), Basic life (metabolic processes with respect to evolutionary trajectories), and finally from a general frame of Physics-chemistry.

The map allows us to make important empirical distinctions, for example, allowing us to render the links between Alice’s vocal gesture, mood, and current family situation under the forms-of-life and Self frames, and the dynamics of two adults coordinating interaction in conversation under a more generic sensorimotor frame. Thus, as a heuristic, the map allows us to draw particular temporal ranges of interdependent processes and constraints, and move our focus between frames in a manner that best suits the nature of the phenomena under scrutiny: we can move to the left of the map, for example, to make considerations only with regards to the statistical regularities of human bodies in a species-specific ecological niche. Alternatively, one can move to the right of the map to investigate the (re)-organisation of interdependent processes and constraints at the level of particular persons or specific activities, such as Alice’s conversation with the therapist. In this way, the mapping strikes a balance between nomothetic and idiographic approaches.

In summary, we propose a heuristic mapping of organising frames to explore how people time-range in a meshwork of interdependencies. More generally, the map presents the idea that (human) living is simultaneously determined by multiple organising frames. Each frame represents a degree of emergence and enablement of complex activity.

## The Backbone: Constraint Interdependence

Organising frames can be distinguished by their nested rank and temporal range, but *what exactly is* organised within each frame? We need a fine-grained and bottom-up approach addressing the *kind* of organisation involved when we talk about organising frames. The answer we propose consists of seeing inter-timescale relationships as comprising multitudes of constraints on processes in a temporal range.

The key idea that connects the map of organising frames with the notion of constraint is that *networks of constraints constitute forms of emergent organisation that manifest degrees of collective interdependence*.^10^ For example, the map represents the basic domain of life as contained within distinctive temporal boundaries: life’s maximum temporal range spans from the slowest timescales of evolution to the fastest timescales of molecular bonding in metabolism. What this framing means is that the maximum temporal range of life contains networks of interdependent constraints that sufficiently account for the maintenance and emergence of life from the wider domain of physics. Constraints lying beyond the timescale extrema of life constitute life’s necessary material conditions but do not form interdependencies with the constraints properly emergent within life.^11^ Similarly, the temporal ranges sufficient to describe a particular personal trajectory contain only a subset of the timescales of life. A single person’s life trajectory is populated by constraints tightly interdependent in a much narrower frame to which external constraints lying on very slow or very fast timescales of evolution and metabolism constitute its boundary material conditions.

### From Independent to Interdependent Constraints

Independently considered, constraints are seemingly prosaic: while being sat on a chair, the chair’s solid surface opposes the gravitational trajectory of your body towards the ground beneath; the chair’s surface is a constraint. This basic limiting aspect is extrapolated in the metaphorical use of the word constraint, such as when one says that budgetary limitations constrain an architectural design. However, when examined closely, constraints do not just limit, they also allow something else to happen (Juarrero, 1999). Doors can pivot on one side because of the constraining action of hinges attached to door frames; hinges and door frames are constraints that *enable* swinging doors. Such constraints are thus simultaneously reducing degrees of freedom and making an otherwise less likely behaviour more likely to happen.

Constraints allow non-spontaneous changes to happen by channelling energy into fewer degrees of freedom (Kauffman, 2008). Doors that are not attached to door frames will not swing from one side spontaneously with the wind. Conversely, with hinges in the right places, the kinetic energy of the wind is transferred into the few degrees of freedom that allow a particular, otherwise unlikely, rotational motion. An example of making the unlikely become much more likely is what enzymes do in metabolism. Enzymes are constraints by virtue of channelling the thermodynamic flow of a process into an alternative pathway that increases the process’s rate of reaction. Given the presence of enzymes, reactions that would otherwise take too long to occur (and thus are non-spontaneous), take just the right amount of time within the temporal range of metabolism.

Crucially, any constraint is always degrading at some timescale, yet the timescale of degradation of the constraint and the relevant timescale of the constrained process are necessarily different. The capacity of constraints to act on processes depends on constraints being statistically stable structures in a temporal-relative way. For example, from the relative point of view of the constrained processes, enzymes remain unchanged^12^ – similar to how chairs and hinges are stable from the point of view of the ecological scale of sitting people and swinging doors. What matters is that constraints are not consumed in the process on which they act and thus maintain their *temporal symmetry* with respect to the constrained process (Moreno and Mossio, 2015). This symmetry occurs either by means of keeping a stable structure (a hinge), or maintaining a constant flow of fast degrading structures with similar and replicable properties. Accordingly, there is nothing in principle against the possibility of finding fast-degrading constraints acting on slower processes.

**FIGURE 2:**
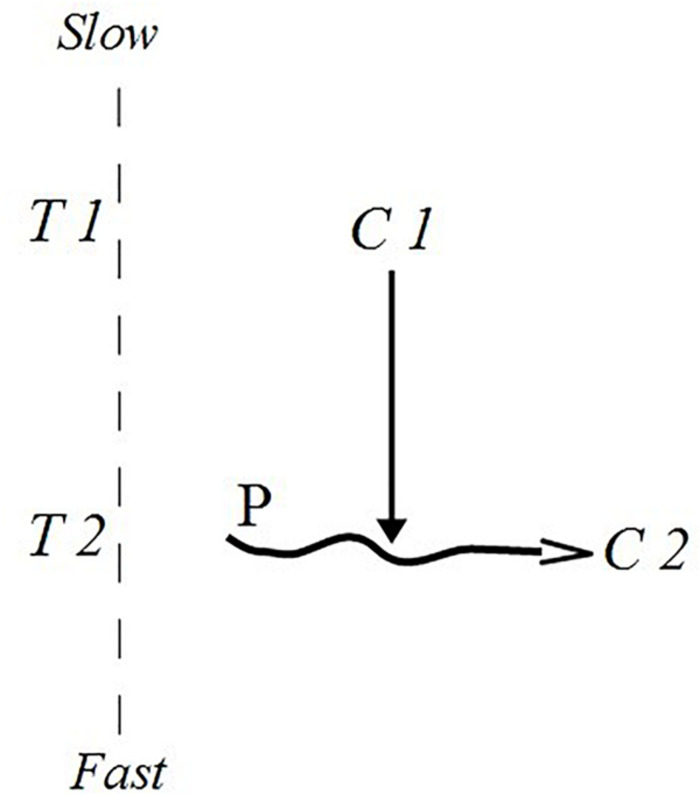
Constraint dependence. Based and adapted from Montévil and Mossio (2015). Notice that the horizontal dimension in the graphic representation does not represent chronological time “moving” in some direction; thus, the arrow of processes P only represents the production and maintenance of constraint C2, not time.

Systems biology theorists Montévil, Mossio, and Moreno point to crucial properties of sets of constraints.^13^ By hypothesis, they propose that any two constraints may constitute *mutual dependency* (Montévil and Mossio, 2015; Moreno and Mossio, 2015). Their crucial observation is that biological constraints such as enzymes are constantly maintained, repaired, and replaced by non-spontaneous processes that require the existence of enabling constraints at other timescales. In this way a direct dependence of at least two constraints can be established without implying any form of exchange of energy/matter between the two. In Figure 2, constraint C2 in T2 (timescale 2) is dependent on C1 in T1 because the process P that maintains C2 is constrained by C1. T1 is a slower timescale relative to T2. We may think of C1 and C2 as being catalysts, whereby catalyst C2 is dependent on the catalytic action of C1 on the process P that regenerates catalyst C2 in T2.

In principle it is possible to find *cycles of interdependency* whereby a chain of dependent constraints closes onto itself: a chain of constrained processes (re-)generates constraints for other processes that in turn generate the constraints at the beginning of the chain. One may think of C1 as being dependent on a series of constraints that link back to C2, which is itself dependent on C1. Montévil and Mossio call this loop “constraint closure” Montévil and Mossio (2015). Crucially, a distinction between *fast constraints* and *slow constraints* is necessary to make this loop work. It must be possible for processes belonging to the cycle to be affected by constraints maintained at faster timescales.

At the physiological range, fast constraints can be found in processes of growth and remodelling of tissue in which the slow (re-)shaping of a tissue is under control of fast constraining flows.^14^ In anticipation of subsection “Replicable Constraints in Conversation”, vocal gestures (“utterances”) are seen as instances of “replicable constraints” (Pattee and Rączaszek-Leonardi, 2012). In the terms of the constraint dependency logic, such replicable constraints can operate as fast constraints on other processes in interpersonal interaction. In any case, fast constraints are populations of similar structures that manifest statistical properties that warrant their constraining action at the relevant timescale.

**FIGURE 3:**
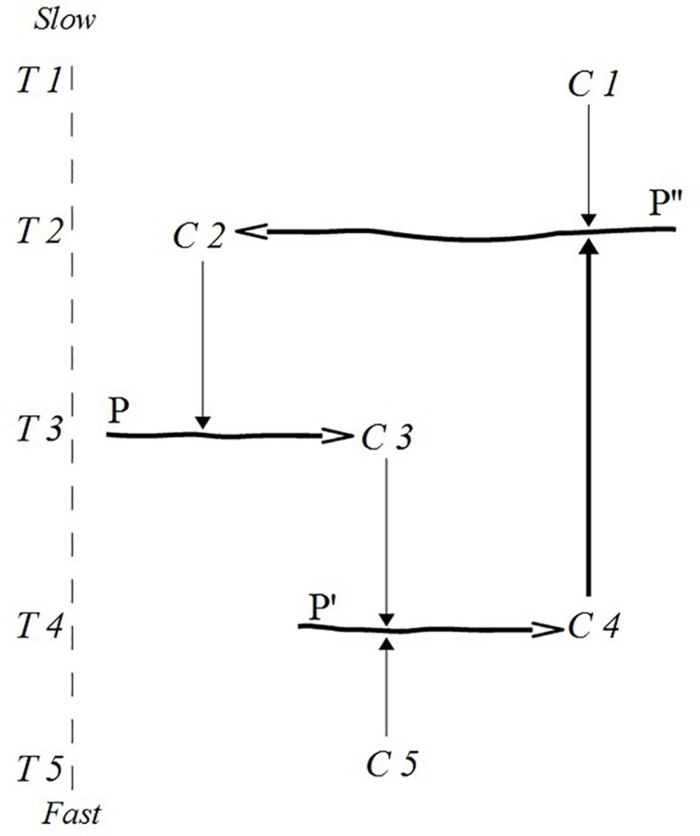
A constraint cycle constituted by two slow (C2, C3) and one fast constraint (C4). Based and adapted from Montévil and Mossio (2015).

Thus, logically, *cycles of constraint interdependence are achieved by the heterogeneous linkage of slow and fast constraints*. This logical requirement is a way of bringing light to interactions between timescales far apart in a temporal range. Since cycles of constraint interdependence occupy temporal ranges they cannot be reduced to single timescales.^15^ In Figure 3, the process P″ that maintains constraint C2 in T2 is constrained by C4. C4 is a fast constraint that needs constant replicating flow in T4 in a way that renders it with statistically similar properties at T2. The whole cycle includes only C2, C3, and C4, and their slow and fast dependencies in a temporal range consisting of at least three timescales (T2, T3, and T4).

### How Do We “Scale-Up” Constraint Closure?

Going from enzymes to vocal gestures, or from cells to the global society (Chavalarias, 2020), requires a careful unpacking of how we can scale-up the constraint closure formalism. Being an organisational principle, with scale-invariant properties, constraint closure allows for implementations in multiple systemic levels. For example, it has been recently applied to mapping glycemia regulation (Bich et al., 2020). Likewise, Nunes-Neto et al. (2016) use constraint closure to map interactions between plants, soil microorganisms, and pollinators in large ecosystems.

Nevertheless, we do not scale-up in the conventional sense of going from “small to large” (e.g., from cell to tissue, to organ, to organism, etc.) or from “fast to slow”; instead, we use the mapping of organising frames (Figure 1) to go from general to specific. What this means, starting from the basic life frame, is that we go from a wide range of metabolic and evolutionary networks of constraint interdependencies into narrower frames that manifest deeper entanglements at “mid-range” timescales – timescales of everyday human living. As we narrow down and move from strict metabolic organisation to the complexity of social life and personal histories, the concept of closure needs to be carefully calibrated to softer and more fluid interdependencies at play in those frames. Montévil and Mossio (2015) suggest that on empirical grounds, constraint closure should be interpreted as a matter of degrees of intensity, or better, as a *tendency towards constraint closure*. We use this idea to transition from the kind of hard constraint interdependencies present in the basic-life organising frame to the softer interdependencies of the forms-of-life and Self frames. We could think of each of the organising frames as domains in which a tendency towards constraint closure may be expressed with different degrees of intensity. In what follows we show different degrees of intensities of constraint interdependencies with the use of the notion of affordance.

#### Affordances and Constraints

From a relational perspective, affordances are action-relevant relations between organism and environment (Chemero, 2009). In the case of chairs, for instance, there is a relation between the material stability and regularity of chairs, the statistical regularities of human bodies and movement (also called effectivities in canonical EP), and the histories of coupling agent-environment under particular socio-material conditions – the history of a particular person in a community in which chairs are regularly manufactured and used for sitting. The three relata in this account – regular chairs, regular bodies, and the biases in social practices – can be seen through the lens of constraint theory. Firstly, the dynamics of sitting behaviour P1 at timescale T1 is constrained by the two slow constraints C1 (regularities of chairs) and C2 (regularities of bodies). Secondly, beyond the timescale of sitting behaviours, C1 and C2 are maintained by slower (and faster) processes associated with the physical stability of materials (the chair) and the self-generating processes of the body. Thirdly, the third relatum, the social niche and practice, brings a meshwork of processes and constraints in wider temporal ranges. On the one hand, an agent’s history of interactions undergirds the particular biases of the fast endogenous dynamics of their central nervous system and body. In the skilled intentionality framework, these fast changes correspond to fluctuating states of action readiness. These states amount to experiences of tension and a tendency towards optimal grip in the situation by responding (or not) to the sitting affordance (Frijda, 1986; Bruineberg and Rietveld, 2014). On the other hand, a person’s current participation in social practices belongs to slow timescales of change and has its own inertia manifested in a myriad of regularities in the inherited social niche, such as the geographical places in which chairs tend to be present, how they tend to be located in space according to particular uses, etc. Crucially, some of these aspects can be seen as constraints binding together to produce the saliency of an invitation to sit in a given situation, including particular habits and other idiosyncratic regularities that play a role in modulating selectivity and responsiveness, and determine the invitational character of a particular affordance (see Heft, 2001, 2013). For example, the constraint of ritual sitting in religious services acts on an individual’s socially acquired attunement and responsiveness to using chairs.^16^

With a more radical version of affordances seen as temporally extended phenomena, van Dijk and Rietveld (2017) tackle the problem of acting in anticipation of complex sociomaterial events. They discuss how multiple affordances at fast timescales can be intertwined and nested within larger affordances at slow timescales. They bring examples from artistic and architectural projects that can take several months to complete; projects that not so much implement a prefigured plan as unfold diachronically (Ingold, 2013). In their view, a project such as building a shed for the next harvest season is constituted by a large-scale affordance (e.g., the prospect of having a shed ready for the harvest), which sets up the conditions for its own continuation by constraining the available smaller affordances at shorter timescales, for instance, using a particular tool for cutting timber for the walls of the shed. In turn, engaged participants would guarantee the continuation of the larger project by means of acting with respect to the small-scale affordances that emerge in the activity. Converging into a particular complete product, actions – such as cutting planks to a size that fits with other planks already in place – become gradually more interdependent and constrained. As the construction process moves towards its final stages, participants are pulled into a narrower set of possible solicitations from affordances. For example, in late stages of construction it becomes less inviting to use a large hydraulic excavator.

In our view, van Dijk and Rietveld (2017) support an unorthodox temporality-based view of affordances.^17^ The authors capture a form of circular dependence that unfolds in the improvisatory performance of the activity and funnels itself into a particular subdomain of possibilities.^18^ In our interpretation, the circularity corresponds to constraint cycles whereby slow-timescale trajectories selectively constrain fast-timescale dynamics, and the pursuit of fast-timescale action selectively channels slow-timescale dynamics.

### Unpacking the Ecological and Enactive Connections

Affordances may be complex meshworks of fast and slow constraining relationships criss-crossing organisms and the socio-material environment. We benefit from using the concept of affordance, broadly defined, to exemplify the use of the logic of constraints in human-relevant temporal ranges,^19^ yet this beneficial connection is bidirectional.

The framework introduces a precise notation capable of capturing inter-scale relationships (e.g., between events, rhythms, deadlines, etc.) and may be able to show how affordances are maintained by constraint interdependencies within an organising frame. Accordingly, the mapping of organising frames (Figure 1) can be used to clarify distinct types of affordances based on the underlying constraint cycles from which affordances derive their regularity.^20^ We may be able to define: (a) species-generic affordances, (b) affordances related to conventions and the socio-cultural niche, and (c) the emergence of idiosyncratic affordances based on personal histories. In brief, external and lawful relations between stable properties of the environment and the animal’s effectivities are reinterpreted in terms of the correspondence between constraints, at very slow phylogenetic and material timescales, acting simultaneously on the (fast) ecological timescales of behaviour. Very slow constraints manifesting law-like properties belong to extremely wide temporal ranges that include phylogenetic and speciation timescales in the sensorimotor frame. Likewise, moderately slow constraints are associated with social conventions in the Forms-of-life frame (Bruineberg et al., 2018), and personal idiosyncrasies belong to narrower temporal ranges of the Self frame.

The connection with EA is most evident in the light of the continuity thesis of mind and life.^21^ For EA “the organisational properties distinctive of mind are an enriched version of those fundamental to life” (Thompson, 2007, p. ix). The framework we propose is precisely based on the idea that the organisational properties of sets of interdependent constraints can be found across all organising frames, from basic life to instances of human interaction, for example, psychotherapy.

An important common ground with EA is the effort to seek the basis of the organisation of life in a balancing act precariously played on the edge of imminent material decay (Di Paolo and Thompson, 2014). Biological constraints are neither infallible nor law-like; they decay irreversibly unless processes catalysed by other constraints replenish them. The more nuanced insight offered by the constraint perspective is the crucial distinction between constraint closure and process closure (Moreno and Mossio, 2015; Kauffman, 2019). The latter, unlike the former, is constituted necessarily by a chain of transformations of matter/energy that closes onto itself, whereby the material outputs of one process become inputs for the next one and a closed loop is achieved. Constraint closure, however, does not require a chain of matter/energy transformation; it only requires that each localised constraint is maintained by a particular process of transformation of matter/energy.^22^ If such a local process fails, the associated constraining capacity deteriorates (Deacon, 2013), and a series of failures may propagate in the constraint loop, not because a pipeline of transformations of energy/matter fails but because the series of constraining actions fail to occur. In short, constraint closure, and more generally constraint interdependence, although fully grounded on particular material dynamics, does not constitute a closure of the underlying processes themselves.

This subtle but crucial distinction allows us to link widely heterogeneous kinds of processes spread across broad temporal ranges by virtue of their local constraining capacities. For example, we can link vocalisations and other behaviours, not by virtue of how matter/energy is transformed (air molecules in movement do not cause other motor activations), but by virtue of how they are organised as a cycle of constraining actions. In what follows we finally sketch how to take the logic of constraint cycles to the realm of human conversation. We briefly unpack the conversational present (see Figure 1) in a way that serves the analysis of the case study in section “Time-Ranging in Psychotherapy: An Ethnographic Investigation”.

### Replicable Constraints in Conversation

Vocal, manual, and facial gesturing of persons in conversation configure particular structures in a flow that has constraining effects in the current moment. For any skilful individual, these structures manifest the fine-tuned entrainment of vocal tract, hands, and facial musculature in a way that allows them to be reproduced quickly and economically at fast-paced behavioural timescales. In the flow of a conversation, vocal and other types of gestures constitute the locale of constraining activity within a much larger meshwork of constraints at slower and faster timescales.

Following the constraint-view in ecological linguistics (Pattee and Rączaszek-Leonardi, 2012; Steffensen and Harvey, 2018; van den Herik, 2018), utterances are behavioural events that operate as *replicable constraints*, reducing the degrees of freedom and harnessing an interactional dynamic. Crucially, in the logic of constraint closure, replicable constraints satisfy the necessary condition of the existence of fast constraints within constraint cycles.

**FIGURE 4:**
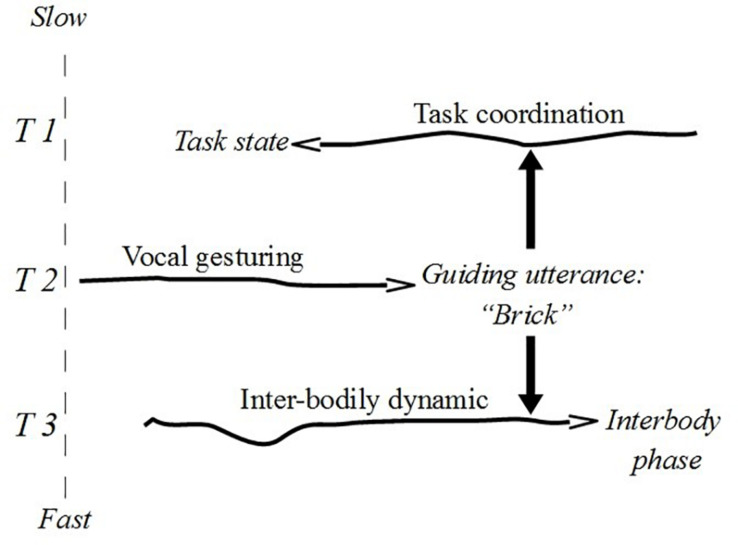
Guiding utterances as replicable constraints.

Utterances, moreover, can be characterised both as fast and slow constraints relative to the component of interactive dynamics on which they have constraining effects. For example, when Wittgensteinian builders yell “brick”, “pillar”, and “slab” in order to orient the attention of a fellow builder, their linguistic actions take place at a timescale between faster interbodily sensorimotor coordination (grabbing and moving stones) and slower paced task/work coordination. The linguistic activity is responsible for the production of discernible changes in the medium (air)^23^ that bear on the interbodily sensorimotor dynamic and on the task coordination. Figure 4 shows these double constraining actions (fast and slow) with the constraint notation.

**FIGURE 5:**
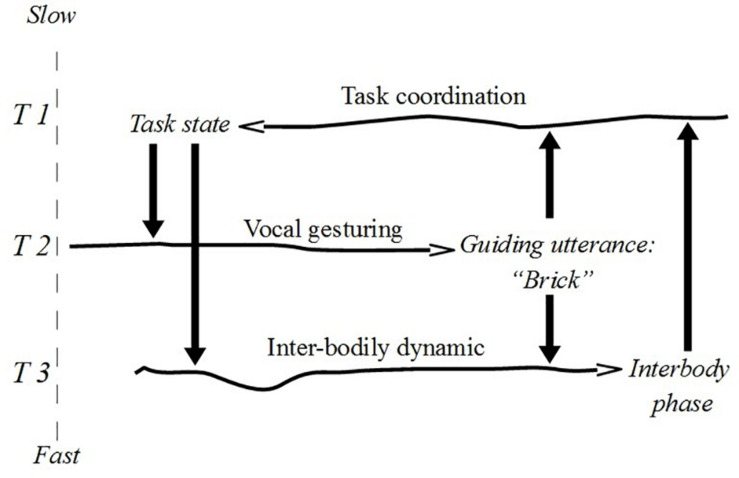
Interlaced constraint cycles.

The covariance between vocal gesturing and task coordination can be made explicit by singling out mutually dependent constraints between these processes. Assuming a fast constraining action on task coordination, we can now characterise a slow constraint looping back on vocal gesturing. In this case we may think of particular states or phases of task coordination as constraints reducing the degrees of freedom of linguistic attentional orientation. Call this covariance a *tight constraint cycle*. We show this cycle in Figure 5 (the cycle in T1–T2) interlaced with another cycle describing the close relation between particular phases of fast interbodily dynamics and the states of task coordination (the cycle in T1–T3).^24^

By singling out fast-produced constraints that manifest statistical similarity across iterations, we are able to show how sets of constraints fold onto themselves to form constraint cycles. The covariance between features of body-to-body entrainment and the use of vocal gestures can be interpreted in an economical way through the multiscalar constraint cycle logic. However, it is clear that dialogical interaction is underdetermined by such a tight cycle. Indeed, the covariance does not tell us much about how vocal gestures can have the specificity of its constraining effects in the first place (Steffensen and Harvey, 2018).

**FIGURE 6:**
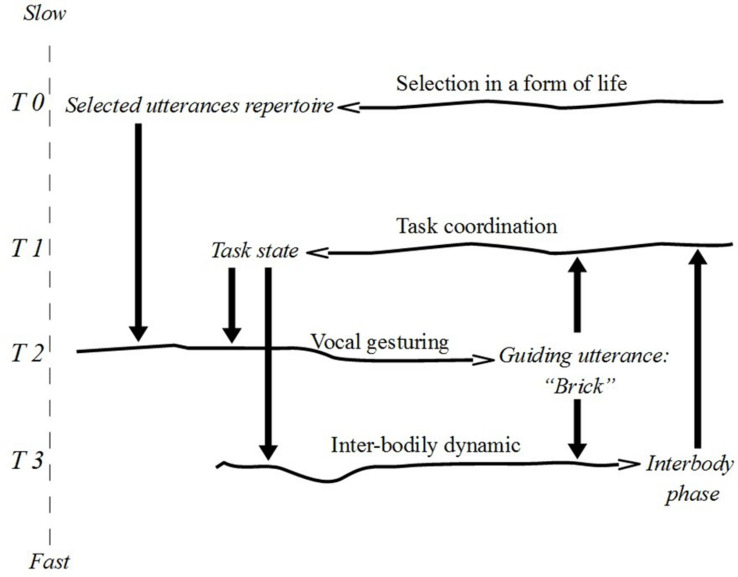
Constraint cycles and slow constraint.

To ask how vocal gestures become constraints is to ask how the unlikely synergy that regenerates and maintains specific vocal gestures becomes more likely; that is, we need to ask what other constraints are in place operating on linguistic activity (and on the tight cycle). We point to social linguistic practices (in forms-of-life frame) that select a repertoire of utterances as a source of constraints (specific selection and distribution of utterances) on vocal gesturing (Pattee and Rączaszek-Leonardi, 2012). This is depicted in Figure 6.

At this point, we can only sketch other possible slower constraint dependencies, for example, social practices that calibrate attention, slow changing moods, roles and membership in family relationships and social practices, etc. (van den Herik, 2018). Crucially, we suggest that the task of the observer is thus to interpret and establish the maximal temporal range necessary to lay out a satisfactory number of constraints that collectively manifest constraint interdependence. There are no special kinds of constraints that can do the explanatory work. The task of mapping meshworks of constraints demands real-life examples.

## Time-Ranging in Psychotherapy: An Ethnographic Investigation

We propose that one interpretation of the aim of psychotherapy is that it helps patients develop time-ranging skills in disentangling and re-entangling constraint cycles of stable, but dysfunctional and painful, patterns of behaviour. The empirical questions of the analysis thus amount to: How do patients become skilled time-rangers? How is cognitive-emotional change established in a conversational present in a way that allows patients to integrate changes in their bio-social systems?

In this section, we investigate how time-ranging skills are practised by emphasising the role of embodied languaging (Fowler and Hodges, 2011; Thibault, 2011; Steffensen, 2013, 2015; Jensen, 2014; Cowley, 2019). In essence, by eschewing mentalist models of psychopathology and psychotherapy, we trace psychological change processes to the increased mastery of time-ranging and the recalibration of constraint cycles in psychotherapeutic interactions. We argue that attention-guiding and perceptual learning, as well as guided interoception, are crucial resources for patients’ self-reflexive sensitivity towards painful behavioural patterns.

We explore these themes by investigating a single case from an ethnographic dataset consisting of video and audio recordings of all therapy sessions for 24 patients in psychotherapy. The data were collected as part of the research project *The Ecology of Psychotherapy: Integrating Cognition, Language and Emotion*, conducted at the University of Southern Denmark. The data were collected at an outpatient clinic for patients with anxiety disorders, obsessive-compulsive disorders, and personality disorders at a Danish psychiatric hospital.^25^ The analysis is guided by the conceptual model of MT presented in previous sections as an explanatory framework for demonstrating how time-ranging emerges in psychotherapy. Methodologically, the analysis relies on a qualitative-idiographic framework, as known from cognitive ethnography (Hutchins, 1995; Steffensen, 2013; Trasmundi, 2020) and multimodal interaction analysis (Goodwin, 2018).

The case involves a female patient in her mid-thirties (pseudonymised as “Alice”). Alice is treated by an experienced female therapist (at about the same age) with expertise in mentalisation-based treatment (Bateman and Fonagy, 2016). Alice was referred to the outpatient clinic after hospitalisation because of a long-lasting major depression and severe panic disorder. After her initial assessment, she was diagnosed with agoraphobia with severe panic disorder, and with recurrent depressive disorder currently in remission. Alice has suffered from lifelong conflictual interpersonal relationships, and she experiences serious relational problems with her spouse and her mother. She is easily agitated, and a recurrent theme in the therapy is how this agitation manifests.

### Speech Rate Between Embodied Behaviour and Social Systems

**FIGURE 7:**
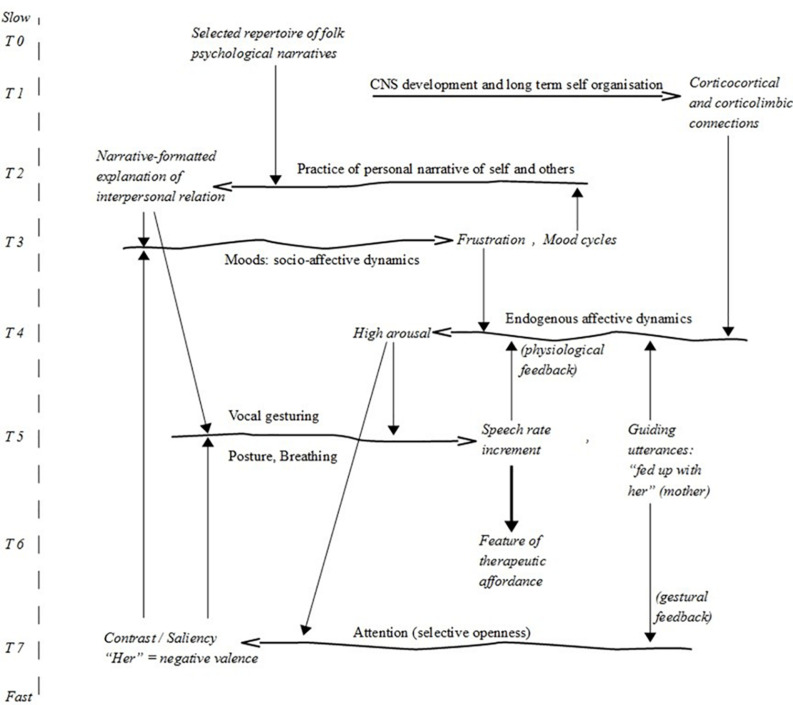
Constraints cycles *before* the intervention. For simplicity, we do not show the organisational frames shown in Figure 1. T4–T7 pertain to the conversational present. The specific constraint dynamics are extensively discussed in the text. Note the status of the constraint “speech rate increment” as a feature of the therapeutic affordance.

**FIGURE 8:**
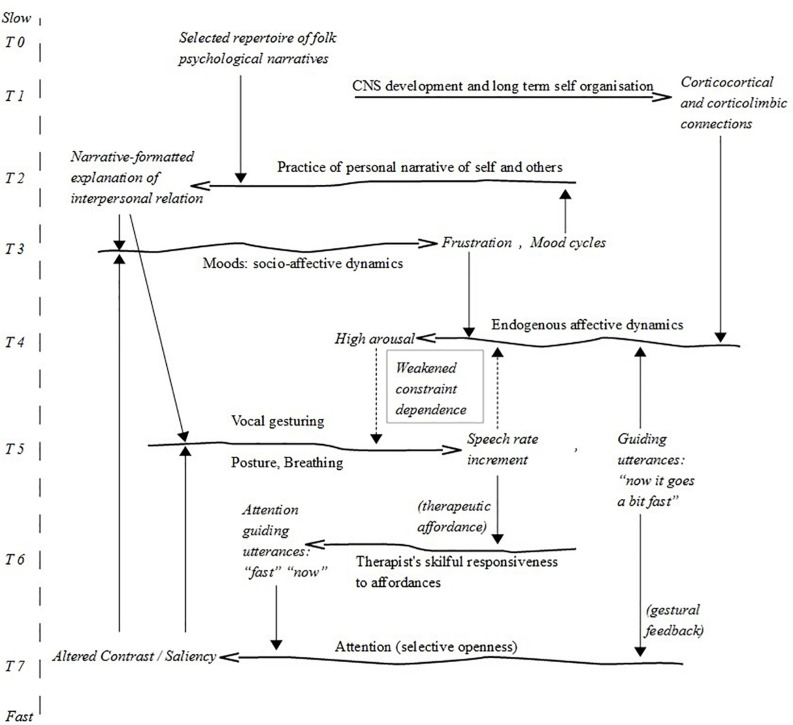
Constraint cycles during the intervention. The therapist’s attentional guidance alters Alice’s selective openness to her own vocal gesturing. This new constraint link (via the therapist’s work) weakens the constraint interdependency between increased speech rate and high arousal (square in the middle).

We explore Alice’s case by taking a starting point in a phenomenon that appears repeatedly in the dataset: the therapist picks out an increase in Alice’s *speech rate* (i.e., number of phonetic or phonological syllables per second). Pertaining to the conversational present, speech rate is an easily observed phenomenon. Apart from idiosyncratic variations and context/genre differences, it depends on the speaker’s emotional arousal (Siegman, 1985). However, rather than assuming that arousal is an unobservable phenomenon in the head/mind of the patient, we follow James (1983) in assuming that arousal *is* an embodied phenomenon, which includes accelerated speech rate, breathing, and facial and manual gesturing. It is this tight coupling between arousal and accelerated speech rate that justifies our focus on speech rate as a core phenomenon within the conversational present (see T5 in Figures 7, 8).

Given her dual role as an observer and participant, the therapist can intervene in the interaction as soon as she observes relevant changes in Alice’s behaviour, including her speech rate. These interventions take a highly standardised form across the dataset^26^ :

*so I notice that you begin to speak significantly faster* (session 5; timecode 23:05)

*I- I start noticing that whoops now it begins to go fast* (session 8; 13:08)

*whoops now- now it begins to go fast again* (session 8; 27:49)

*I notice that ehm it goes fast today* (session 11; 12:50)

*it goes fast right now* (session 14; 33:24)

*I notice that you begin to talk a bit fast* (session 17; 15:07)

To exemplify the implications of the interventions, we highlight the first occurrence in session 5. This intervention follows a 14-s long patient narrative, where Alice produces 91 phonological syllables, that is, a speech rate of 6.5 syllables per second.^27^ This speech rate is significantly higher than elsewhere in the interaction. For comparison, the first time the patient produces a coherent narrative (2:01 min into the session), Alice’s speech rate is 4.6 syllables per second, and in a similar narrative 2 min before the therapist intervention (i.e., 20:07 min into the therapy), her speech rate is 4.6 syllables per second. In other words, the therapist reacts to a sudden increase in Alice’s speech. Immediately after the intervention, Alice’s speech rate drops to 5.6 syllables per second (measured in her first segment longer than 10 s), and half a minute later it has further dropped to 5.3 syllables per second. Ten minutes after the intervention, Alice’s speech rate is 5.0 syllables per second, and hence almost back to the initial rate. A similar pattern is found in the other examples listed above.

Crucially, by pointing Alice’s attention to her speech rate, the therapist in fact prompts Alice to change it. This change is an instance of time-ranging. Before the intervention, we observe a constraint cycle between arousal and speech rate, where arousal constrains speech rate in a tight feedback loop (T4–T5 in Figure 7). In the intervention, a new constraint cycle is established between Alice’s arousal and speech rate and the therapist’s expressed observation (T4–T5–T6–T7 in Figure 8). This constraint cycle is conditioned by the therapist’s skilful responsiveness to vocal gesturing and behavioural changes (“feature of therapeutic affordance” in T6) dependent on the cycle of speech rate-arousal co-variation. From an ecological point of view (van den Herik, 2018), language functions as a perceptual tool. Thus, the therapist’s expressed observation constrains Alice’s attention (T6–T7 in Figure 8), which in turn constrains the speech rate, allowing Alice to weaken the constraint interdependence between her high arousal and her speech rate increment.^28^

Therapy has an overt focus on helping the patient regulate their emotions. Generalising from this example, we may hypothesise that patients appropriate a way of regulating their emotions by learning to notice, and in turn to modify, a feedback loop between a given emotional pattern and a specific behaviour in the conversational present. The change observed in this case consists of Alice’s weakening the constraint interdependence between her arousal and her increased speech rate whilst preserving (although altered) the coupling between processes of attention, vocal gesturing, and affective dynamics.

Both constraint cycles pertain to the entangled organisational frame of the conversational present. However, these local dynamics are embedded in constraint cycles that pertain to other organisational frames. Thus, if we zoom out, we observe that the increase in arousal is inextricably bound up with the content of Alice’s socio-affective dynamics (in T3) and a practice of personal narrative (T2). Consider Excerpts 1 and 2, in which the therapist’s observations are embedded:^29^

**Excerpt 1: session 5; timecode 23:05 – 23:13**

T: so I notice that you begin to speak significantly faster

P: yes

T: yes (.) and you say yourself

P: [but it is because it is something] that is difficult

T: [that you feel irritated]

P: yes but it is because

T: [yes]

P: [it] is difficult for me

**Excerpt 2: session 14; timecode 33:24 – 33:37**

T: it goes fast right now

*P: yes (.) but it’s because I get madly irritated (.) and then I get a bit frustrated that I get this thought every time we discuss (.) I’m fed up with her* [i.e., Alice’s mother]

These two excerpts suffice to demonstrate that the constraint cycle between arousal and speech rate in turn is constrained by Alice’s feelings of frustration due to her mother. Alice’s enacted arousal is constrained by emotions pertaining to her position in a larger (non-local) social system (Steffensen, 2012): *I’m fed up with her*. By taking Alice’s social relations into consideration, we move beyond the conversational present and into the organisational frame of forms-of-life (cf. Figure 1). Thus, we observe a new tight constraint interdependence between Alice’s high arousal and a stabilised pattern of socio-affectivity that pertains to Alice’s social system (T3–T4 in Figures 7, 8). However, it is not simply the case that these emotions build up in the patient’s social system (at an “everyday timescale”) and surface in the dialogical system within the conversational present. The relation allows for a reciprocal dynamic where the emotional reactions are investigated in the dialogical system. In other words, by modifying her way of attending to the non-local socio-affective dynamics, Alice can alter the pathological constraint cycle between the social system and the dialogical system (the large cycle T3–T4–T7 in Figure 7). Again, a key factor is a change in attention, which is brought forth by the therapist’s use of languaging as a perceptual tool.

To determine a complete network of constraint cycles, we should consider how Alice’s personal narrative practices are present in the dialogical system, whereby narrative-formatted utterances habitually trace Alice’s emotional dynamics (be it in the social system or in the dialogical system) in a way that orients attention to a longer timescale of Alice’s childhood experiences and trauma. An example is found in the following excerpt from session 8:

**Excerpt 3: session 8; timecode 02:18 – 03:53**

P: I don’t know how to […] like mm with all those (.) with the neglect I felt from my mother when me and <SPOUSE> started to date and (.) when she found out (.) that we should have <CHILD> and (.) the neglect from my dad when I was very young and such things.

On the one hand, these autobiographical narrative elements concern the frame of Alice’s Self, that is, how she accounts for her past, which in turn is an organising frame that enables how she experiences and interprets the conversational present. On the other hand, it relates to the sensorimotor frame, that is, how she has developed a set of embodied reactions in her childhood, which impact on her adult behaviour, both in her social systems and in the dialogical system.^30^

While we cannot go back in time, we can observe how legacies of childhood events manifest in Alice’s real-time embodiments in part constrained by a stable neural architecture (T1–T4). We see that in relation to the therapist in the conversational present, as well as in her renditions of earlier encounters with her spouse. Thus, the past is part of the patient and not detached from her present embodied behaviour: there is a constraint cycle between her anxiety as a habituated bodily response (since early childhood within the Sensorimotor frame), the persistent presence of her mother in her current social system (within the Forms-of-life frame), and her behaviour in the conversational present. This temporal entanglement is an important aspect of the therapeutic work, for instance by weakening these constraint interdependencies by attending to the stabilised patterns in other organisational frames that underlie Alice’s behaviour. In the next section, we will explore how Alice and her therapist do so by engaging in time-ranging.

### Time-Ranging and Perceptual Learning

In section “Speech Rate Between Embodied Behaviour and Social Systems”, we established the constraint cycle entanglement outlined in Figure 8. Further, we have argued that therapeutic progress is achieved by weakening the tight constraint interdependencies through extended constraint cycles that involve the therapist guiding Alice’s attention. In this section, we will explore time-ranging as a reconfiguration of constraint cycles. We take a starting point in Excerpt 4:

**Excerpt 4: session 8; timecode 27:49 – 28:01**

T: whoops now- now it begins to go fast again

P: yes

T: can you feel that

P: yes haha

T: we just entered the irritation there

P: yes

T: yes (.) so can we- can we just- if you should take me back to when you left from here last time

P: yes

The therapist’s first and last utterances exemplify time-ranging techniques. As previously discussed, the first utterance follows a sequence in which Alice gets carried away by her frustrations over her mother. By sharing her observation about the increased speech rate, the therapist prompts Alice to time-range by attending to her situated behaviour. The last utterance has a somewhat different dynamic: having established that the speech rate is caused by the patient entering a difficult emotional space, the therapist requests that they jointly explore the difficult emotion connected to past events in the social system. Following Conversation Analysis, we consider the initial phrase *so can we- can we just-* as an example of *repair*, that is, “a set of practices designed for dealing with the types of difficulties which emerge in talk” (Liddicoat, 2011, p. 208). In this case, the repair indicates that the therapist acknowledges that staying in the difficult emotions is somewhat troublesome for Alice, which is exactly why she tries to escape them. By eliciting Alice’s acceptance to stay focused on the difficult emotions, they collectively attend to Alice’s traumatic past, but now not only from Alice’s vantage point, but from the vantage point of a distributed cognitive-emotional system. Thus, the dialogical system is effectively a distributed cognitive system where the therapist provides the trustful context necessary for Alice’s exploration of the past. In other words, by relying on an experienced time-ranger (an emotional Sherpa, so to speak), Alice can re-appropriate her past, and hence change her present (social and dialogical) situation.

Such time-ranging dynamics are a crucial aspect of psychotherapy. However, given the long-term goal of psychotherapy, it is obviously insufficient that Alice is capable of engaging in time-ranging when guided by the therapist. For therapy to work, Alice must develop her time-ranging skills further, so she can monitor and moderate her behaviour and her emotional reactions outside of therapy. Due to the nature of the dataset, we have no data on such behaviour outside the therapeutic setting, but we do have a glimpse that indicates that Alice’s self-reflexivity changes throughout the course of therapy. The context of Excerpt 5 is that Alice is engaged in a rather agitated narrative about her relatives, when suddenly she interrupts herself in the first turn in the excerpt:

**Excerpt 5: session 10; timecode 27:11 – 27:19**

P: now it goes a bit fast

T: yes that-

P: [yes]

T: [that] you could hear

P: yes

T: [yes]

P: [haha]

T: that was very well done that you noticed that (.) because I noticed too that I got detached

It is striking that the typical “therapist formulation” (picking up on the increased speech rate) is uttered verbatim *by Alice* (Alice’s own guiding utterance in T5 in Figure 8). Alice appropriates time-ranging skills by emulating the therapist’s perspective. She observes and attends to her own speech rate, which is what the therapist usually does. She thus exhibits what Eleanor Gibson called perceptual learning (Gibson, 1969): “Perceptual learning is the process whereby perceptual information becomes increasingly differentiated and specific to the things in the world and to what one can do with those things” (Adolph and Kretch, 2015, p. 130). While the paradigm case of perceptual learning is infant development, learning to differentiate between levels of speech rate, and learning to associate them with different levels of arousal is a crucial skill. Alice’s perceptual learning hinges on her ability to pick up differences in her own speech rate. Alice develops her ability to register the emotional dynamics, established through past interactions, that constrain her embodied, situated behaviour. Thus, the perceptual learning *vis-à-vis* her own embodied behaviour, becomes a tool for registering how past events still play a role in her present situation. By learning to detect the signals of how the body reacts to the memory of these past events, we can learn to detect the past as a temporal pattern in the present. In short, Alice learns to *feel* how she feels – through reflexive self-perception and the ability to link it to organisational frames beyond the locally perceived reality.

Alice engages in increased speech rate behaviour on multiple occasions after this episode, so while we have seen the initial enskilment, we have not seen the habituation of that behaviour. However, therapy is a non-linear phenomenon, so it is unsurprising that this skill is not developed once and for all. Further, it must be kept in mind that this case study pivots on a specific behavioural pattern, the perception of increased speech rate. The perceptual learning associated with self-reflexivity, can be directed towards all kinds of behaviour, and given our restricted focus we will not be able to detect a general increase in self-reflexivity.^31^

### Time-Ranging as a Transformation of the Constraint Cycle Network

So far we have established how the interactional noticing of speech rate functions as a time-ranging device, and we have argued that developing flexible time-ranging skills goes hand in hand with the possibility of using the dialogical system as a distributed cognitive-emotional system (cf. Hollan et al., 2000). Thus, we have shown how Alice engages in the dialogical system to act more reflexively and less habitual in the social system. Thus, by appropriating the therapeutic organisation of perceptual learning, Alice engages skilfully in time-ranging, making her more sensitive to her bodily reactions and behaviours in the dialogical system, and potentially also beyond. The crucial question to be raised here is how this time-ranging skill allows Alice to explore new dimensions of her psychopathology.

To answer this question, we take a starting point in the therapist’s speech rate observation in session 8 and trace the kind of time-ranging triggered by it:

**Excerpt 6: session 8; 13:08 – 13:27**

T: I- I begin to feel whoops, now it begins to go fast

P: yes

T: and I think when was it that- that it began to go fast (.) do you have a sense of that

P: I think it did so when I should tell about my mother and the- what she

T: her role in [it or]

P: [her role] in it yes

T: that’s where it began to go fast (.) okay

Alice’s observation that accounting for her mother’s role in her traumatic past triggers the accelerated speech rate sets off a deeper reflection, not on the temporal range of her childhood, but rather on the temporal range of her recent *reflections* on her childhood:

**Excerpt 7: session 8; 13:29 – 13:27**

P: but I think that the reason why my mother has also begun to- it’s because it is only recently that I, like- I have always blamed my father always

T: what have you [blamed him for]

P: [that it has always been] his fault

T: yes

P: well, that he has beaten us and my mother and (.) whatever he has done

T: that he has beaten you

P: yes

T: that he has [xxxx]

P: [that he has] yes that he has subjected us to the things he did, well I have never seen my mother play a role in that (.) but the more I like talk to people about it and stuff, where they say, well your mother has also played a role in it she has- she is like the one who should protect you

There are two crucial observations to be made. First, Alice accounts for a para-therapeutic change in her recent past, which has made her reconsider her mother’s role in her childhood. While we have no way of ascertaining if this change is constituted in or beyond prior therapeutic encounters, it is clear that Alice experienced an important change process in-between an autobiographical and a social systemic timescale. Second, in her account for this recent insight, Alice opens with two noticeable self-interruptions: *but I think that the reason why my mother has also begun to- it’s because it is only recently that I, like- I have always blamed my father always*. In the first instance (*my mother has also begun to-*). Alice interrupts herself just before she ascribes blameworthy agency to her mother. In the second incident, she interrupts her narrative about her recent feelings for her mother by recurring to a more stabilised narrative structure of her father’s culpability. In both cases, Alice reveals an inhibitory mechanism that prevents her from uttering critique of her mother. This mechanism has become a crucial part of her Self frame, and for that reason, it is connected with many upsetting feelings when she, later in the therapy, considers breaking up with her mother. Interestingly, while this inhibitory mechanism is at play, Alice also opens a self-reflexive space where she can in fact explore the vulnerability of how she has held her mother inculpable for her own struggles. We get a further glance into this vulnerable space in Excerpt 8, which follows 3 min after the previous excerpt.

**Excerpt 8: session 8; 16:39 – 17:02**

P: I get so angry that she at all- that he is (.) such a swine and he th- (.) can take the liberty- well, he thinks he can take the liberty to treat other people like that (.) but that my mother she (.) () that she (.) could let it happen haha that she had children with him (.) that she didn’t (.) end it earlier on

Here we see a micro-transformation from the inhibitory pattern (cf. the self-interruption in *I get so angry that she at all-*) to an explicit critique: *but that my mother she (.) () that she (.) could let it happen*, where the micropauses indicate that it is a difficult perspective for her.

This emergent pattern shows us that Alice enacts a deeply entangled triad of constraint cycles through which her conversational Self reflects on how her social Self has developed a more nuanced view on her childhood Self. Alice’s time-ranging thus allows for polyphonic reasoning where she recruits multiple voices (Linell, 2009) and perspectives in her struggle to understand her past. This polyphonic reasoning is prompted by the therapist’s modulation of the narrative temporality, and it indicates that the overall constellation of constraint cycles is itself transforming in a way that allows Alice to develop a more nuanced self-reflexivity.

In summary, by weakening the constraint interdependence between fast speech and high emotional arousal through modulating attention, the therapist prompts the system to explore other possible constraints available in a larger frame of forms-of-life. What Alice implicitly finds is that she can introduce a new narrative to her way of telling the personal story. In Excerpt 7, she starts with an acknowledgement of her usual story (“*I have always blamed my father always*”), before she moves on to a novel story line: “*she is like the one who should protect you*”. In Excerpt 8, she effectively *integrates* the two story lines by connecting them: “*he thinks he can take the liberty to treat other people like that*” leads directly to “*she (.) could let it happen haha that she had children with him*”.

What Alice gains here, it seems, is a new story line that allows her to keep track of why she has ongoing relationship issues with her mother. This change of perspective is achieved through the following steps: (1) weakening the constraint interdependence (by relying on the therapist’s attention orientation), (2) exploring new constraints (narratives available in forms-of-life), and (3) creating a new constraint interdependence (a narrative-formatted explanation of why she’s angry with her mother).

Crucially, the second step requires an access to a public repertoire of narrative-formatted folk storytelling (Hutto, 2008) at the population level in the Forms-of-life frame. This particular format consists of agents that behave with unfairness (the father), patients that receive mistreatment (the mother and Alice), as well as patients who end up being agents who act wrongly by omission (the mother in relation to Alice). Persons learn these formats through media (e.g., novels, etc.) and other people’s stories. It is a crucial dimension of psychotherapy that it involves *deep time-ranging*. It involves not just the personal Self frame but *exploratory acts* in the Forms-of-life frame as well.

### Time-Ranging and Embodied Memory

We have shown how time-ranging allows Alice to explore her embodied behaviour in the conversational present, in relation to the slow processes that constitute her social system, as well as to her Self (as narrative) and her traumatic autobiography. In terms of Figure 1, this time-ranging unfolds in the interplay between the three organisational frames of the conversational present, the Self, and the forms-of-life. However, as it is clear from Figure 1, our overall theoretical framework predicts that the sensorimotor frame constrains Alice’s psychopathology as well. This assumption is fully in line with both EP and EA, in which there is a rich history of tracing emotions to embodied experiences (Colombetti, 2013).

In this last part of the analysis, we explore the sensorimotor embodiment of Alice’s time-ranging. It comes to the fore when Alice is prompted to explore how her autobiography is not only narrated but crucially also *experienced* as a situated embodied sensation. In terms of Figure 8, the behavioural continuity on T5 is not just in terms of speech, but crucially also a question of posture, breathing, and interoception.

We see that in Excerpt 9, which surfaces shortly after Excerpt 8. Alice recounts an episode that her mother has told her, in which Alice’s father violently assaulted her mother, when she was pregnant with Alice. As expressed in Excerpt 8, Alice is angry, which prompts the therapist to explore the embodied sensation of her anger:

**Excerpt 9: session 8; timecode 17:23 – 19:24**

T: () Alice (.) what is it that you feel right now (.) when we talk about it

P: well it is that feeling of unreality (.) I just come to think (.) well she even fears for her life (.) but she chooses to have kids with him anyway

T: what happens in your body right now (.) ()

P: mm (.) that- th- that I cannot explain (.) I cannot explain it

T: no

P: a bit hopeless I think

T: ()

P: I mean

T: breath a while

P: yes (.) yes

T: yes

P: and I do think

T: do try to stay here

P: yes haha

T: you are drifting away

P: yes haha

T: yes (.) try to tell me how it feels like

P: I’ve got a dry throat

T: you got a dry throat

P: or a dry mouth

T: yes

T: how do you feel in your shoulders

P: I don’t know- I can’t feel that

T: you can’t feel them

P: I can’t feel shit

T: how does it feel right here

P: I don’t know

T: how ()

P: so shallow

T: [shallow]

P: [it all]

P: yes

T: yes (.) how do you breathe

P: shallowly (.) I think

T: shallowly

P: not all the way down with my stomach

T: no (.) so when we talk about (.) your childhood and the things that happened (.) to your mother and to you (.) then it becomes very unreal

P: yes

T: it becomes something that has happened to another

P: yes

T: and it is as if you disconnected

P: yes (.) but I have to consider it in order to find out why I am like I am

T: okay

P: I think (.) and perhaps under- perhaps under- also get to understand why I suffer from anxiety and why I am so (.) vulnerable and in general a very troubled person

Strikingly, Alice reports that she is unable to feel her bodily reactions when prompted by the therapist: *I can’t feel that, I can’t feel shit*, etc. By confirming a feeling of being disconnected, Alice is confronted with a time-ranging barrier: rather than re-experiencing her own autobiography, *it becomes something that has happened to another* – in the words of the therapist. The trauma is present in the local here-and-now, and not just a past event long ago. Or vice versa: the body is irreducible to a present state, it is its past too. On this deep sensorimotor level, time-ranging appears in Alice’s disability to feel her shoulders, her breath, her body. Thus, her time-ranging is dysfunctional because she is unable to integrate her bodily reactions with her self-reflexivity in the conversational present.

In this excerpt, we see an important dimension of psychotherapy, as the therapist picks up on the embodied aspects of Alice’s time-ranging. By prompting her to feel parts of her body, she helps Alice to realise that she cannot feel her body as she invokes her traumatic past. On this account, *guided interoception* functions as a time-ranging technique that allows patients to explore the sensorimotor experiences that are part of the psychopathological entanglement. Further, the therapists asks two questions (“*it becomes something that has happened to another*”) and (“*it is as if you disconnected*”), that functions as interpretational frames for Alice’s interoception, so that they contribute to her self-reflexive understanding of how the horizontal stretch of temporality curls into a present body-with-a-history.

## Discussion

The case study shows how the framework of MT allows us to single out particular instances of constraining action (e.g., changes in speech rate, changes in narrative content) for purposes of analysis whilst taking seriously the necessity of building a bigger picture that does justice to the interdependencies criss-crossing the boundaries of brains, bodies, and environment. Those boundaries, although existing in nature, are secondary for the framing of the proposal, instead, our starting point is in the middle of the changing meshworks of processes that realise mutual constraining interdependencies. With the guide of the proposed mapping, the observer is prompted to locate particular relationships on the map by moving, not so much inward/outward across the traditionally assumed boundaries of the brain and body (Ramstead et al., 2019), but “up”/“down” across layers of timescales, temporal ranges, and organising frames.

The particular outlook afforded by the framework coincides with important aspects of experience. Thus, rather than standing alone as a seemingly abstract theorisation, the framework speaks of aspects of lived temporality just as they appear not only in the experience of psychotherapy but also in everyday life: persons converse in the shops, write their memoirs, or make works of art, with a spontaneous sense of deep temporality and the self-reflexivity brought forth by time-ranging. The concept of time-ranging is proposed to capture the experiential signature of MT: the lived richness and depth of field of the present moment populated by non-local events and what appears to be absent in the here-and-now.

Persons gain the skills of (re)configuring the coherence of the here-and-now by meshing multiple temporalities. For example, by using vocal gestures that bring forth constraint interdependencies to bear on the situation, persons live a rich present as they navigate the constraint landscape in flexible ways, positioning themselves within long-term projects and established relationships (cf. Steffensen, 2015).

We show how time-ranging manifests in the professional skills of psychotherapists and in instances of therapeutic progress. Firstly, psychotherapists are capable of picking up and pointing to a series of relevant gestures from the patient and trace their interdependence with slower-than-conversation timescales of the patient’s changing participation in family life, career, public life, intergenerational relationships, and beyond, into population-level patterns and narratives. Secondly, with exploratory actions accompanied by the therapists, persons may learn to notice and then reconfigure the coherence of their here-and-now by reorganising and bringing other constraint interdependencies to the fore, thus transforming their agency and their ability to anticipate future events, that is, becoming more flexible time-rangers.

With the framework, the idea that individuals navigate in a thick here-and-now thanks to a history of interactions acquires a sharper rendition. Moreover, it brings together under the same light both the ecological legacies and organismic habits belonging to a deep history of individuals and populations, and the quick changes at short timescales responsible for the variability and flexibility of particular interactions. Each of the organising frames proposed connects long timescales of legacies/habits and short timescales of variability.

Crucially, the proposed distinction between slow and fast constraints within a temporal range brings light to the synergy between long-term habits and the capacity to explore new unprescribed possibilities. Fast constraints, which vocal gestures instantiate, may provide a key to understanding the mutability of interactions. Iterations of fast constraints are never identical, thus, by harnessing the improvisatory nature of articulatory gestures – as fast constraints – individuals may pull new constraint interdependencies to bear on the present situation. Slow constraints allow change by virtue of the existence of larger repertoires of regularities, in the case of psychotherapy, in the form of substitutive or complementary narratives of personal relationships, existing in a public storytelling domain, that can be incorporated anew into the patient’s own interpretations of the present situation. In this way, the unique synergy of fast and slow constraints accounts for both the (re)production of behaviour and the emergence of genuinely new configurations of constraint interdependencies. This synergy is a more precise reason for analysing interdependent networks of constraints rather than observing constraints in isolation.

### Further Discussion: Ecological and Enactive Connections

We elaborate on the need of a fresh new look at conceptualisations of temporality in the EP and EA literature. MT, as a third perspective, brings a different light to these concepts.

The concept of time-ranging incorporates aspects of the “ecological-enactive” view offered by the “skilled intentionality framework” (Rietveld and Kiverstein, 2014). Following the skilled intentionality framework, time-ranging implies that persons need to be minimally sensitive, selectively open, and ready to act towards reducing the felt tension in a situation. In other words, a basic form of skilled intentionality needs to be in place whereby actions tend towards an optimal positioning in a “field of affordances” (Rietveld and Kiverstein, 2014). The notion of tendency towards an optimal grip captures this phenomenon (Merleau-Ponty, 1945/2002; Bruineberg and Rietveld, 2014). In a situation, through sedimented histories of experiences, persons find themselves being pushed and pulled into particular actions, positions, and trajectories, usually leading to a temporary “improved grip in the environment” (Bruineberg and Rietveld, 2014). Merleau-Ponty’s basic example is the “microscopic” adjustment and positioning of the enthusiastic art gallery visitor near the optimal distance to a picture (Merleau-Ponty, 1945/2002, p. 352). In the current proposal, we have elaborated on how persons optimally position themselves in temporal terms, reconfiguring constraint interdependencies with the use of actions that seek to reduce the felt tension in a situation.

We have also shown how part of the canon of EP can also be translated into the perspective proposed. The work of the therapist is to guide attention and thus expand the repertoire of temporal ranges that are meshed in the present situation. We have argued for an interpretation of the therapeutic work in terms of perceptual learning and education of attention.

More interestingly, we leave open the discussion regarding a fruitful connection with the theory of affordances by showing how affordances manifest different historical depth in terms of their associated constraints. As we have pointed out in section “Unpacking the Ecological and Enactive Connections”, what seem to be contrasting views between different interpretations of Gibsonian notions are seen, we propose, as based on a difference in what is assumed to be the fundamental extent of the temporal range of phenomena under scrutiny. Similarly, the distinctions between organising frames may parallel contemporary distinctions between physical world, habitat, and *Umwelt* (Baggs and Chemero, 2019), thus, potentially bringing temporal depth to the evolving ecological literature.

The framework of MT remedies the current poverty of concepts with regards to how human living spills over many timescales. EA correctly notes how dynamics unfold in what can be called a “thick here-and-now” (Buhrmann et al., 2013, p. 13). This expression conveys the idea that the current moment is not simply a narrow window constituted only by those actualised states of a system. Instead, “The current state reflects a history of changes that the system has undergone over time. In this way, the totality of past events is brought to bear on the current situation” (Buhrmann et al., 2013, p. 13). The thick here-and-now is thus a necessary consequence of the strong dynamicism present in EA and its focus on the histories of interactions (Cummins and de Jesus, 2016), that is, the established tendencies of the agent(s)-environment coupling, its “virtual traces”, and “virtualities” (Di Paolo, 2015). However, while EA acknowledges the importance of “complex temporal and intensity conditions such as speeds, deadlines” (Di Paolo et al., 2018, p. 28) as well as “different rhythms, temporal scales, and phenomena of synchronization and co-variation […] at the core of [a living system’s] constitutive processes” (Barandiaran et al., 2009, p. 379), it falls short of offering a toolkit for addressing and tracing these temporal phenomena in a concrete way. The aim of this article is to remedy this lacuna with a combination of a mapping of temporal ranges, organising frames, and an expanded conceptualisation of the constraint closure formalism offering an economical way of accounting for the structuring effect of histories of interactions.

The constraint closure formalism by Montévil and Mossio (2015) has various unique characteristics that may be complementary to EA’s notion of individuation. Sets of constraints that form a constraint closure collectively maintain and stabilise one another across a wide range of spatiotemporal scales without additionally requiring strict closure of underlying processes. We have developed this idea further by proposing networks of interdependent constraints that necessarily occupy wider temporal ranges and do not fit squarely into traditional boundaries of the brain, body, social groups, and environment. In our interpretation, these properties allow us to flexibly narrow down or open up the scope of temporal ranges under consideration. Accordingly, rather than stipulating *a priori* and singling out the existence of essential constraint closure structures, we propose to use the mapping of organising frames to investigate domains with different intensities of tendencies towards constraint closure. By doing so, the framework could deal with different research interests and smooth the conceptual transition between nomothetic and idiographic perspectives: from theoretical biology to the analysis of particular personal histories (e.g., in psychopathology).

Finally, by proposing a tendency towards constraint closure in wider temporal frames we leave open the discussion of the conditions and constituents of normativity (bio-social norms) (Steiner and Stewart, 2009). The effect of a tendency towards constraint closure implies that activities and situations channel themselves into forms of path dependence and habits that manifest norms generated within the domain itself of habitual interactions (James and Loaiza, 2020). Likewise, in EA, habits display the requirement of self-maintenance that is needed for an account of normativity (Barandiaran and Di Paolo, 2014; but see a critique in Barrett, 2017). Our view may elaborate further the notion of habit and is inclusive of the varieties of bodies view of EA (Di Paolo, 2020). The framework, however, is not exhausted by conservative views that ground normativity essentially in body-bound individuality, self-production, and self-distinction.^32^

### Future Work

We have shown how the proposed framework occupies a space not only in between but also beyond EP and EA, while facilitating the conversation across the table. Although we do not foresee a unified “E” theory – a marriage of EP and EA – the framework shows promising connections between E approaches and recent attempts to develop a distributed perspective on language that takes a starting point in meso-scale organism-environment interactivity (Cowley, 2011, 2012; Thibault, 2011; Steffensen, 2015; Harvey et al., 2016; Thibault and King, 2016; Steffensen and Harvey, 2018; Gahrn-Andersen et al., 2019), as well as wider anthropological discussions of social life in “E” terms (Loaiza, 2016, 2019; James and Loaiza, 2020). Many details of these connections are, however, still missing. Future work could also focus on a more robust methodology of analysis, for example, by linking to other existing non-reductionist views in mental disorder research. In particular, it is possible to see how the theory of constraints could be complemented with the network model of mental disorders (Borsboom, 2017). While this article has contributed with a proof of concept for the viability of taking a starting point in MT, we hope that future work will pave the way for a broader methodological applicability.

## Data Availability Statement

All datasets generated for this study are included in the article/Supplementary Material.

## Ethics Statement

The studies involving human participants were reviewed and approved by Danish National Committee on Health Research Ethics. The patients/participants provided their written informed consent to participate in this study.

## Author Contributions

JL proposed the hypothesis in “The Backbone: Constraint Interdependence” and “Discussion”, and the overall outline. SS designed the study (see acknowledgments). SS and ST collected data, analysed data, and identified the case. JL and SS wrote the final draft. ST contributed to earlier drafts, provided input, comments, and suggestions that were implemented in the final drafts. All authors contributed to the article and approved the submitted version.

## Conflict of Interest

The authors declare that the research was conducted in the absence of any commercial or financial relationships that could be construed as a potential conflict of interest.

## Footnotes

1 An important difference with classic EP is the more liberal take on the notion of affordance. In Bruineberg et al. (2018, p. 6.): “the overwhelming majority of affordances available in the human social relations are not lawfully specified by the energy in the environment, but are determined by conventions, customs, practices, or other regularities”.

2 Although the current framing of time-ranging incorporates some of the phenomenological insights of the “skilled intentionality framework”, as elaborated by Bruineberg and Rietveld (2014), the original introduction of time-ranging in Cowley and Steffensen (2015) does not rely on those insights. Instead, the original version brings elements from the interactivity and distributed language literature.

3 Elements of the two themes are familiar to the reader with a background in EP and/or EA. The characterisation itself of two themes, one experiential and the other organisational re-elaborates EA’s dual phenomenological and operational perspectives. We signal the appropriate links with existing EP and EA ideas along the text and summarise the connections in the discussion.

4 Trasmundi and Steffensen (2016) discuss one such example where an emergency medical team orients to habitual solutions in order to decide on the future line of action *vis-à-vis* a patient.

5 An expression used by EA theorists to describe the historicity of agents (Buhrmann et al., 2013, p. 13).

6 The example is taken from Cowley and Steffensen (2015, p. 478).

7 See an EA approach to reflexivity in de Haan (2020).

8 There is a rich embodied cognition and EA body of literature that emphasises the importance of phenomenal temporality in psychotherapy (e.g., Kyselo and Tschacher, 2015; Tschacher et al., 2015a,b; Fuchs, 2018). For instance, Fuchs (2018) argues that linear time is a result of measurements of physical events, whereas temporality of life emerges as cyclical processes that often manifest in subjective whole-bodied experiences. He argues, in line with our approach, that such manifestations are the result of an experience that is being re-enacted again and again. However, while Fuchs – and colleagues – emphasise lived time, or the temporality of the body, our approach extends this view by providing a model that traces temporality to multiple temporal ranges that constrain personal development. See also Enfield, 2014.

9 The logic of the map is based on “specification hierarchy” (Salthe, 1991).

10 Many constraint-based views crop up across various fields of scholarship that seek to explain so-called “higher” cognition while circumventing traditional explanatory concepts, such as reference, representation, content, and semantic information. A constraint-based view shows up in current versions of radical embodied cognitive science (Bruineberg et al., 2018) as well as in various theories of linguistic action (Pattee and Rączaszek-Leonardi, 2012; Steffensen and Harvey, 2018; van den Herik, 2018). Other influential constraint-based views have been formulated previously by Ashby (1961), Polanyi (1968); Juarrero (1999), Deacon (2013) amongst others. Constraint interdependence, based on the idea of “constraint closure” (Montévil and Mossio, 2015; Moreno and Mossio, 2015; Kauffman, 2019), is however, a novel addition to the constraint-based literature within ecological and radical embodied approaches.

11 For example, the distance of the earth to the sun constrains life to follow a particular evolutionary path, but it is not interdependent with the processes generated within life. Such distance is an external independent constraint.

12 Not significantly changed. In other words, enzymes do not significantly transfer energy to or become consumed by the process they constrain; thus, they degrade at a different timescale.

13 Based on the work of Stuart Kauffman, Robert Rosen, Howard Pattee, and Alicia Juarrero.

14 Montévil and Mossio (2015) point to the slow process of bone mineralisation constrained by the (fast constraint) enzyme alkaline phosphatase.

15 The word *cycle* thus refers to a logical form, not to cyclicality on a single timescale.

16 We leave out a detailed characterisation of the example with the use of the constraint notation.

17 van Dijk and Rietveld (2017) use the idea of temporality mainly to substantiate the claim that affordances are always anticipatory regardless of timescale (van Dijk and Withagen, 2016), we propose propose to use temporality itself for a characterisation of affordances that involves unpacking the range of relevant timescales.

18 This view, however, seems incomplete and needs a discussion of what Stuart Kauffman calls the “adjacent possible” (Kauffman, 2019, Kauffman, 2000) at the root of innovation – the emergence of completely novel solutions, situations, trajectories, etc. (cf. Cowley and Markoš, 2019).

19 We, however, do not uphold the dependence of the proposed framework on the notion of affordance. As we show in section “Time-Ranging in Psychotherapy: An Ethnographic Investigation”, the investigation of constraints in a real-life case study promises to be, strictly speaking, a self-standing theory. We thank a reviewer for pressing us to clarify this connection.

20 By mapping interdependent constraints in wide temporal ranges this proposal can be part of an effort to connect slow evolutionary processes and processes of niche construction in social practice. As has recently proposed (Heras-Escribano, 2020), linking niche construction with natural selection brings the theory of affordances closer to a grander extended evolutionary synthesis (Heras-Escribano and de Jesus, 2018).

21 There is a history of productive exchanges between autopoietic EA (e.g., Thompson, 2007) and the work of Moreno, Mossio, and colleagues. The history of the “constraint” approach and the development of constraint closure theory extends more than 20 years. See an early example in Ruiz-Mirazo and Moreno (2004). The intricacies of the history, parallelisms, and differences between EA’s autonomy and constraint-based perspectives, however, exceed the current proposal. Both are theories of organisational closure (see review in Moreno and Mossio, 2015) and manifest a scale-invariant logic, organisational rather than mechanistic, that can be extrapolated to domains beyond metabolism. We thank one of the reviewers for pressing us to clarify this connection.

22 Clearly, the distinction is not whether we are dealing with open thermodynamic systems. All instances are thermodynamically open far from equilibrium. A process/work cycle requires exergonic (“energy releasing”) and endergonic (“energy absorbing”) processes linked in a chain of energy/matter transformation (Kauffman, 2000); a constraint cycle only requires that exergonic and endergonic processes are present in a way that maintains the constraints necessary for a particular organisation.

23 “Articulatory gestures (Goldstein and Fowler, 2003; Fowler, 2014), highly regularized synergies among the various components of the articulatory tract whose repetition results in auditorily, visually, kinesthetically, and acoustically similar events. [These] resources function normatively in that adult humans regulate their micro-scale vocalizations to match the appropriate patterns and instantiate the appropriate synergies. They achieve this by means of rich phonological memory, entrenchment, sensitization, and related mechanisms” (Harvey et al., 2016).

24 We are not showing other possibilities of cycles, for example, how interbodily coordination in terms of body distance and alignment in a conversation may alter the flow of linguistic activity.

25 All patients enrolled in the research project were asked to volunteer for a project that aimed to understand “how language and body dynamics influence the therapeutic situation”. The patients were informed about the data collection, protection, and pseudonymisation. The patients did not receive any payment or compensation for their participation in the project. The patients were recruited for the project by their therapist during a pre-therapy start-up meeting. All patients and therapists have given written consent to participate in the study, including being the subject of analysis in a research article. Prior to the enrolment of patients, the project was reported to the Danish National Committee on Health Research Ethics.

26 See Supplementary Material for original transcriptions in the Danish language. Data translated by the authors.

27 We follow Hilton et al. (2011) in distinguishing between phonological and phonetic syllables. Since our purpose is to compare speech rates at different stages of the interaction, there is no gain in relying on phonetic syllables. We calculate speech rate in a given segment by identifying a segment of at least 10 s during which one participant produces one coherent turn (ignoring minimal response of the other interlocutor). We then count the number of phonological syllables in a normalised transcript within the identified segment.

28 Given the human bodily layout, attention, however, flighty it may be, manifests statistical similarity across iterations, which allows it to function as a fast constraint on behaviour, in this case on speech rate.

29 Here and elsewhere, T refers to the therapist, and P to the patient (Alice). “(1.2)” indicates the duration of pauses in seconds. Pauses less than 0.25 s are indicated with “(.)”. Brackets in adjacent turns indicate sequences that overlap. In later excerpts, minimal response will be inserted in brackets: [T: hmm].

30 In this context, we ignore the sensorimotor and forms-of-life ranges of the therapist. These temporal ranges include, amongst others, both her own embodied reactions in the conversational present and her training. As for the latter, functioning as a psychotherapist requires enskilment and what Goodwin (1994) famously called *professional vision*. Therefore, the educational background of the therapist, as well as her institutional embeddedness (with leaders, colleagues, supervisors, etc.), condition the dynamics. In spite of that, we focus on Alice, as she is the locus of the psychological change process.

31 However, the dataset includes daily responses to the *Therapy Process Questionnaire* (Schiepek et al., 2019). Alice does report a significant improvement in her therapeutic progress and in her self-reflexivity/insights.

32 Similarly, we leave open the discussion about how the notion of autonomy used in EA literature can be complemented, constrained, or even subsumed within the models of constraint closure and the more liberal idea of a tendency towards constraint closure in wider temporal ranges. Accordingly, the notion of autopoiesis (itself a subclass of autonomy. See Maturana and Varela, 1980, 1987; Varela, 1997), may be seen as a dense subset of constraints within the larger network of constraint interdependencies in what we call the basic-life organising frame (Figure 1). The semipermeable membrane of an autopoietic system is an important, but not uniquely special, constraint on the interior reaction processes (Virgo et al., 2009).
