# Supplementary material for: Multiscalar Temporality in Human Behaviour: A Case Study of Constraint Interdependence in Psychotherapy
Source: Front Psychol. 2020 Aug 20;11:1685. doi: 10.3389/fpsyg.2020.01685 (PMC7468424; doi:10.3389/fpsyg.2020.01685)
Supplement: Supplementary file 1 [file Data_Sheet_1.PDF]

Excerpt 0

Danish Originals

0a	så jeg lægger mærke til at du begynder at snakke væsentligt hurtigere (session 5; timecode: 23:05)
0b	je- jeg begynder at mærke hov nu begynder det at gå stærkt(session 8; timecode: 13:08)
0c	hov nu- nu begynder det at gå stærkt igen (session 8; timecode: 27:49)
0d	jeg lægger mærke til at øhm at det går stærkt i dag (session 11; timecode: 12:50)
0e	det går stærkt lige nu (session 14; timecode: 33:24)
0f	jeg lægger mærke til at du begynder at snakke lidt hurtigt (session 17; timecode: 15:07)

English Translation

0a	so I notice that you begin to speak significantly faster
0b	I- I start noticing that whoops now it begins to go Fast
0c	whoops now- now it begins to go fast again
0d	I notice that ehm it goes fast today
0e	it goes fast right now
0f	I notice that you begin to talk a bit fast

Excerpt 1: session 5; timecode: 23:05-23:13

Danish Original

T: så jeg lægger mærke til at du begynder at snakke væsentligt hurtigere
P: ja
T: ja (.) og du siger selv
P: [men det fordi det er noget] der er svært
T: [du bliver irriteret   ]
P: ja men det er fordi
T: [ja]
P: [det] er svært for mig

English Translation

T: so I notice that you begin to speak significantly faster
P: yes
T: yes (.) and you say yourself
P: [but it is because it is something] that is difficult
T: [that you feel irritated   ]
P: yes but it is because
T: [yes]
P: [it] is difficult for me

Excerpt 2: session 14; timecode: 33:24-33:37

Danish Original

T: det går stærkt lige nu
P: ja (.) men det fordi jeg bliver helt vildt irriteret (.) og så bliver jeg lidt frustreret over at jeg får tanken hver gang vi diskuterer det (.) jeg gider hende [i.e. Alice's mother] ikke mere

English Translation

T: it goes fast right now
P: yes (.) but it's because I get madly irritated (.) and then I get a bit frustrated that I get this thought every we discuss (.) I'm fed up with her [i.e. Alice's mother]

Excerpt 3: session 8; timecode: 02:18 – 03:53

Danish Original

P: jeg ved ikke hvordan jeg skal […] sådan mm med alle de der (.) med de svigt jeg følte fra min mors side dengang mig og <SPOUSE> fandt sammen og (.) da hun fandt ud af (.) at vi skulle have <CHILD> og (.) det svigt min far gav mig da jeg var helt lille og sådan nogle ting.

English Translation

P: I don't know how to […] like mm with all those (.) with the neglect I felt from my mother when me and <SPOUSE> started to date and (.) when she found out (.) that we should have <CHILD> and (.) the neglect from my dad when I was very young and such things.

Excerpt 4: session 8; timecode: 27:49 – 28:01

Danish Original

T: hov nu- nu begynder det at gå stærkt igen
P: ja
T: kan du mærke det
P: ja haha
T: nu gik vi lige ind i irritationen der
P: ja
T: ja (.) så kan vi- kan vi bare lige- hvis du lige skal tage mig tilbage her da du gik herfra sidste gang
P: ja

English Translation

T: whoops now- now it begins to go fast again
P: yes
T: can you feel that
P: yes haha
T: we just entered the irritation there
P: yes
T: yes (.) so can we- can we just- if you should take me back to when you left from here last time
P: yes

Excerpt 5: session 10; timecode: 27:11 – 27:19

Danish Original

P: nu går det lige lidt stærkt
T: ja det-
P: [ja]
T: [det] kunne du godt høre
P: ja
T: [ja]
P: [haha]
T: det var enormt flot at du lagde mærke til det (.) fordi jeg lagde nemlig også mærke til at jeg blev koblet af

English Translation

P: now it goes a bit fast
T: yes that-
P: [yes]
T: [that] you could hear
P: yes
T: [yes]
P: [haha]
T: that was very well done that you noticed that (.) because I noticed too that I got detached

Excerpt 6: session 8; timecode: 13:08 – 13:27

Danish Original

T: je- jeg begynder at mærke hov nu begynder det at gå stærkt
P: ja
T: og jeg tænker hvornår var det lige at- at det begyndte at gå stærkt (.) har du en fornemmelse af det
P: det tror jeg det gjorde dengang jeg skulle fortælle om min mor og den- det hun
T: hendes rolle i [det eller]
P: [hendes rolle] i det ja
T: så begyndte det at gå stærkt (.) okay

English Translation

T: I- I begin to feel whoops, now it begins to go fast
P: yes
T: and I think when was it that- that it began to go fast (.) do you have a sense of that
P: I think it did so when I should tell about my mother and the- what she
T: her role in [it or]
P: [her role] in it yes
T: that’s where it began to go fast (.) okay

Excerpt 7: session 8; timecode: 13:29 – 13:27

Danish Original

P: men jeg tror også grunden til at min mor også er begyndt- det fordi
   det er for nyligt at jeg sådan- jeg har altid bebrejdet min far altid
T: hvad har du [bebrejdet ham   ]
P: [at det har altid været] hans skyld
T: ja
P: altså at han har slået os og min mor og (.) hvad han nu ellers har
   gjort
T: at han har slået jer
P: ja
T: at han har [kidnappet dig]
P: [at han har   ] -ja at han har budt os det har han gjort
   altså det har jeg aldrig kunne se min mor har haft en rolle i (.) men
   jo mere jeg sådan har snakket med folk om det og sådan nogle ting
   hvor de siger jamen din mor har da haft en stor rolle i det hun har-
   hun er jo ligesom den der skulle beskytte jer

English Translation

P: but I think that the reason why my mother has also begun to- it’s
   because it is only recently that I, like- I have always blamed my
   father always
T: what have you [blamed him for   ]
P: [that it has always been] his fault
T: yes
P: well, that he has beaten us and my mother and (.) whatever he has
   done
T: that he has beaten you
P: yes
T: that he has [kidnapped you]
P: [that he has] yes that he has subjected us to the
   things he did, well I have never seen my mother play a role in that
   (.) but the more I like talk to people about it and stuff, where they
   say, well your mother has also played a role in it she has- she is
   like the one who should protect you

Excerpt 8: session 8; timecode: 16:39 – 17:02

Danish Original

P: jeg bliver så gal over at hun overhovedet- at han er (.) sådan et svin og han tr- (.) kan tillade- altså han tror han kan tillade sig at behandle andre mennesker sådan (.) men at min mor hun (.) (   ) at hun (.) kunne lade det ske haha at hun fik børn med ham (.) at hun ikke (.) gjorde det forbi inden

English Translation

P: I get so angry that she at all- that he is (.) such a swine and he th- (.) can take the liberty- well, he thinks he can take the liberty to treat other people like that (.) but that my mother she (.) ( ) that she (.) could let it happen haha that she had children with him (.) that she didn't (.) end it earlier on

Excerpt 9: session 8; timecode: 17:23 - 19:24

Danish Original

T: (   ) Alice (.) hvad er det lige du mærker lige nu (.) når vi snakker om det
P: jamen det er den der uvirkelighedsfølelse (.) jeg kommer lige sådan til at tænke (.) mja hun frygter jo ligefrem for hendes liv (.) men alligevel vælger hun at få børn med ham
T: hvad sker der lige i din krop lige nu (.) (   )
P: mm (.) det- d- d- det kan jeg ikke forklare (.) det kan jeg ikke forklare
T: nej
P: sådan lidt håbløs tror jeg
T: (   )
P: altså
T: træk lige vejret
P: ja (.) ja
T: ja
P: og jeg synes jo
T: prøv bare lige at bliv her
P: ja haha
T: du er på vej videre
P: ja haha
T: ja (.) prøv lige at fortælle mig hvordan mærkes det lige her
P: jeg er tør i halsen
T: du er tør i halsen
P: eller i munden
T: ja
T: hvordan mærkes det i dine skuldre
P: det ved jeg ikke- det kan jeg ikke mærke
T: dem kan du ikke mærke
P: jeg kan ikke mærke en skid
T: hvordan mærkes det lige her
P: det ved jeg ikke
T: hvordan ( )
P: så overfladisk
T: [overfladisk]
P: [det hele]
P: ja
T: ja (.) hvordan trækker du vejret
P: overfladisk (.) tror jeg
T: overfladisk
P: det kommer i hvert fald ikke helt ned i maven
T: nej (.) så når vi snakker om (.) din barndom og de ting der er sket (.) for din mor og for dig (.) så bliver det meget uvirkeligt
P: ja
T: det bliver noget der er sket for en anden
P: ja
T: og det er ligesom om du koblede af
P: ja (.) men at jeg bliver nødt til at forholde mig til det for at finde ud af hvorfor jeg er ligesom jeg er
T: okay

P: synes jeg (.) og måske for- måske for- også få en forståelse af hvorfor jeg har angst og hvorfor jeg er så (.) sårbar og generelt meget bekymret menneske

English Translation

T: (   ) Alice (.) what is it that you feel right now (.) when we talk about it
P: well it is that feeling of unreality (.) I just come to think (.) well she even fears for her life (.) but she chooses to have kids with him anyway
T: what happens in your body right now (.) (   )
P: mm (.) that- th- th- that I cannot explain (.) I cannot explain it
T: no
P: a bit hopeless I think
T: (   )
P: I mean
T: breath a while
P: yes (.) yes
T: yes
P: and I do think
T: do try to stay here
P: yes haha
T: you are drifting away
P: yes haha
T: yes (.) try to tell me how it feels like
P: I've got a dry throat
T: you got a dry throat
P: or a dry mouth
T: yes
T: how do you feel in your shoulders
P: I don't know- I can't feel that
T: you can't feel them
P: I can't feel shit
T: how does it feel right here
P: I don't know
T: how ( )
P: so shallow
T: [shallow]
P: [it all]
P: yes
T: yes (.) how do you breath
P: shallowly (.) I think
T: shallowly
P: not all the way down with my stomach
T: no (.) so when we talk about (.) your childhood and the things that happened (.) to your mother and to you (.) then it becomes very unreal
P: yes
T: it becomes something that has happened to another
P: yes
T: and it is as if you disconnected

P: yes (.) but I have to consider it in order to find out why I am like I am
T: okay
P: I think (.) and perhaps under- perhaps under- also get to understand why I suffer from anxiety and why I am so (.) vulnerable and in general a very troubled person
